# Prescribing Phones to Address Health Equity Needs in the COVID-19 Era: The PHONE-CONNECT Program

**DOI:** 10.2196/23914

**Published:** 2021-04-06

**Authors:** Gill Kazevman, Marck Mercado, Jennifer Hulme, Andrea Somers

**Affiliations:** 1 Faculty of Medicine University of Toronto Toronto, ON Canada; 2 Department of Emergency Medicine University Health Network Toronto, ON Canada; 3 Department of Family and Community Medicine University of Toronto Toronto, ON Canada

**Keywords:** digital health equity, health inequity, digital determinants of health, emergency medicine, COVID-19, public health, health policy, primary care, cell phone

## Abstract

Vulnerable populations have been identified as having higher infection rates and poorer COVID-19–related outcomes, likely due to their inability to readily access primary care, follow public health directives, and adhere to self-isolation guidelines. As a response to the COVID-19 pandemic, many health care services have adopted new digital solutions, which rely on phone and internet connectivity. However, persons who are digitally inaccessible, such as those experiencing poverty or homelessness, are often unable to use these services. In response to this newly highlighted social disparity known as “digital health inequity,” emergency physicians at the University Health Network in Toronto, Canada, initiated a program called PHONE-CONNECT (Phones for Healthier Ontarians iN EDs – COvid NEeds met by Cellular Telephone). This novel approach attempts to improve patients’ access to health care, information, and social services, as well as improve their ability to adhere to public health directives (social isolation and contact tracing). Although similar programs addressing the same emerging issues have been recently described in the media, this is the first time phones have been provided as a health care intervention in an emergency department. This innovative emergency department point-of-care intervention may have a significant impact on improving health outcomes for vulnerable people during the COVID-19 pandemic and beyond.

## Introduction

The COVID-19 pandemic has highlighted many social disparities encountered by vulnerable populations [1]. Vulnerable populations, such as those experiencing poverty or homelessness, have a higher rate of infection and significantly worse COVID-19–related health outcomes [2]. They have been disproportionately affected during the pandemic by a lack of safe housing for physical distancing or self-isolation, difficulty adhering to public health directives, and significant barriers to accessing health care and social services [3].

Emerging digital solutions adopted by health care providers in response to the pandemic rely heavily on phone connectivity and access to the internet [4]. These include^ ^telemedicine, telepsychiatry, and COVID-19–related communications such as testing status updates and contact tracing. Most recently, the Canadian federal government launched a new mobile app for efficient and secure contact tracing, named COVID Alert [5]. Although these new solutions provide exciting opportunities for the future of health care delivery and public health, they may create yet another barrier for those who are unable to afford a phone and phone plan [6]. The inability to access digital services constitutes a newly explored digital determinant of health: digital health equity [7].

A recent study suggests that targeted access to mobile devices can help mitigate the downstream consequences of this health disparity [8]. Given the urgent needs presented during the COVID-19 pandemic, the emergency department (ED) at the University Health Network (UHN), in Toronto, Ontario, responded with a pilot program to provide free prepaid cell phones to vulnerable patients.

## What is PHONE-CONNECT?

Phones for Healthier Ontarians iN EDs – COvid NEeds met by Cellular Telephone, also known as PHONE-CONNECT, is a program founded by UHN emergency physicians to address the health and social needs of vulnerable patients through cellular and digital connectivity. This program was conceived after recognizing that many patients facing digital inequity are at risk for further morbidity despite adequate treatment received in the ED. These risks are compounded by the COVID-19 pandemic wherein contact tracing, physical distancing, and virtual care are heavily reliant on having access to a digital device. The program repurposes donated cell phones and distributes them to eligible patients discharged from the ED as an intervention to address digital health inequity. The aims of the program are to identify people experiencing digital health inequities and address these inequities by improving patients’ access to health care services, information, and social services, as well as to improve social connectivity. Additionally, the program seeks to increase patients’ capacity to follow public health directives (including self-isolation) and their availability for contact tracing during the pandemic.

To the best of our knowledge, this is the first time cell phones have been distributed from EDs to address the needs of vulnerable patients, and the first time cell phones have been distributed from an ED during a pandemic. Since the program’s inception at UHN, PHONE-CONNECT has expanded to include additional Greater Toronto Area sites (ie, St. Michael’s Hospital and Michael Garron Hospital) and academic medical centers (ie, McMaster University).

## Implementation

**Figure 1:**
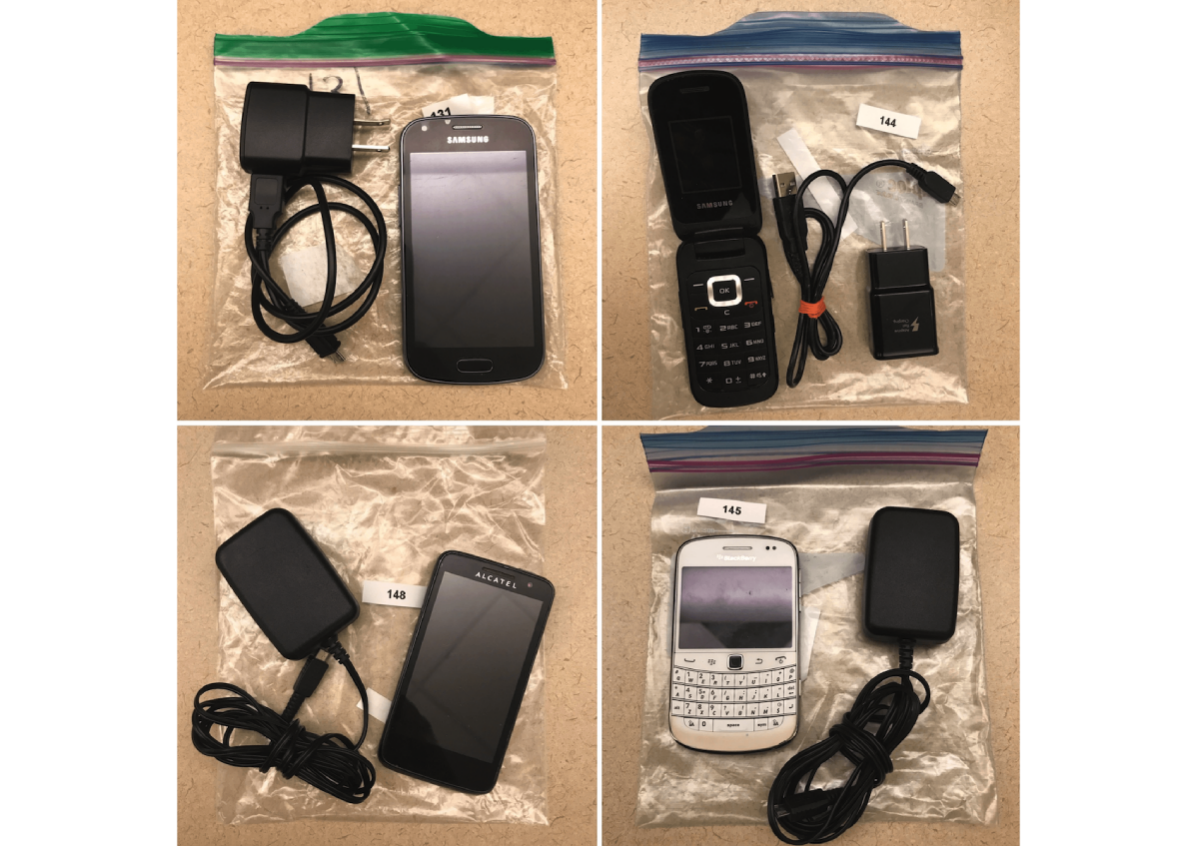
Phone distribution packages.

The PHONE-CONNECT program was initiated with the donation of 50 smartphones and 200 SIM cards with unlimited talk and text features from a telecommunications company. Additional phone donations were sought by word of mouth and via media coverage of the initiative. Members of the public were invited to mail in their pre-used cell phones. Donated phones were checked for operability and compatibility with the donated SIM cards, cleaned of the previous user’s personal information and disinfected. Phone distribution packages (Figure 1) include a cellular device loaded with a SIM card, a compatible phone charger, and a phone manual (when available). A handout explaining the importance of using the phone to connect with health care providers was included, as well as a list of phone numbers for various health and social services.

## Population

Patients are deemed eligible for a phone distribution package if a phone number is not listed on their ED chart. This applies to all patients who do not have access to a telephone and require communication of test results and pending appointments, those who require a phone to facilitate self-isolation, or those who need a device to access follow-up care, virtual or in-person. Health care providers who were trained to administer the phones include physicians, nurse practitioners, physician assistants, registered nurses, social workers, and peer support workers. These providers were informed of the PHONE-CONNECT initiative through daily department huddles, team meetings, and emails. They were given instructions on patient eligibility, administration of phones to patients, and documentation of the patient’s new phone number. The same health care providers were responsible for identifying eligible patients during the course of regular clinical care, orienting the patients to their new phone prior to discharge from the ED, and advising them that they would be contacted for consent for a follow-up interview. Once distributed, the new phone number is added to the patient's electronic medical record, and the patient is encouraged to keep the phone turned on, charged, and available should a health care provider need to contact them. Aside from these requests, the patient may use the phone however they choose.

## Data Collection

The PHONE-CONNECT pilot project is being assessed through an ongoing research study exploring the impact of the phone on the patient’s well-being and their utilization of health care services, as well as the perspectives of the health care providers participating in the PHONE-CONNECT initiative.

Specifically, 2-4 weeks following discharge, research assistants attempt to contact phone recipients through their new device with a maximum of three calls and one SMS text message. A semistructured interview (Multimedia Appendix 1) is then conducted with the phone recipient to explore the role of their new phone in addressing digital health equity. This includes the following: the role of the phone in their ability to self-isolate in the context of COVID-19, their communication with health care providers, their ability to maintain their social networks, and the recipients’ self-reported well-being. Quantitative data collected include the number of phones distributed, the number of participants attending virtual and in-person follow-up appointments, nonattendance to prescheduled appointments, as well as the number of participants tested for COVID-19 and who received their results via the phone intervention.

The impact of PHONE-CONNECT on the distributing health care providers is also being explored. The health care providers are asked to complete a questionnaire using 5-point Likert scale questions assessing their perceptions on how giving a phone in the ED affects their ability to provide care to socially complex patients. Additional questions assess the impact of phone distribution on provider burnout.

## Review of Similar Initiatives

To the best of our knowledge, prior to the COVID-19 pandemic only one program offered free cellular phones for vulnerable populations. In the United States, free cellular phones are offered to a subset of participants in the Federal Lifeline program [9]. A study of people experiencing homelessness in New York City who received free phones through this program found that beneficiaries viewed these free devices as an important tool to help navigate, engage, and ultimately improve their access to care [10]. More recently, and as a response to the digital disparities experienced by the homeless population during the COVID-19 pandemic, two new programs have been described in the media. In the United Kingdom, a program administered by a charity and a telecommunication company was designed to provide 2500 cell phones to eligible recipients living in England, Scotland, and Wales [11]. More locally, the Social Planning and Research Council (SPARC) of British Columbia distributed 3500 smartphones to persons experiencing homelessness at parks and shelters [12].

## Impact

Thus far, 180 devices have been distributed to eligible patients at two sites in Toronto. The personal narratives emerging from our nascent evaluation are compelling and will be highlighted in the publication of a future study. Recipients have used their phones to address their health care needs by connecting with physicians, addictions counsellors, social workers, medical specialists, and Toronto Public Health representatives, and to attend telemedicine appointments. They have accessed suicide hotlines and emergency medical services. They have used their phones to meet their basic needs, by accessing groceries during isolation and securing shelter beds. Furthermore, recipients have strengthened their social networks by virtue of being in regular contact with friends and family.

## Limitations

Despite the reported benefits of providing mobile phones to those experiencing homelessness [10], there are several issues that should be addressed as this program finds greater adoption. Not all patients are inherently “tech savvy” and may incur additional stress and anxiety from using a mobile phone for health-related communication. Appropriate matching of user to device by technical confidence level and familiarity should be considered in the initiation of similar programs. Furthermore, “phone-seeking behavior” could be an unfortunate side effect of this program that may increase ED visits, although this has not been our experience.

Finally, as this is an ED point-of-care intervention, clinical demands on ED providers may increase. Empowering other allied health professionals in the ED to provide phones has addressed this concern. Moreover, our experience suggests that any increased clinical burden may be offset by better ease of ED discharge planning for patients now able to access care beyond the ED.

## Discussion

The need for phone connectivity will increase as the COVID-19 pandemic continues to impact access to health services. We are concerned that if digital equity needs remain unaddressed, vulnerable patients will continue to experience disparate health outcomes of increasing magnitude during the pandemic and beyond. Although further research into the different aspects of digital health equity is necessary for the creation of universal policies and strategies, this innovative initiative is an achievable first step in overcoming the challenge of accessibility. This project may demonstrate that identification of those who are facing this equity issue can be accomplished in the primary care setting. Moreover, a recent study found that improving primary care access via phone connectivity can reduce avoidable ED visits [13]. Therefore, by facilitating access to outpatient health care and social services, the PHONE-CONNECT program may be an innovative solution to reduce unnecessary ED visits or prolonged ED length of stays.

As we continue to pilot this novel ED point-of-care intervention, we hope to further explore the effects of our program on its recipients. We are particularly interested in understanding the effects of this program on access to and utilization of the health care system, social services, COVID-19–related public health interventions, as well as the impact on overall well-being. We are also interested in determining whether the ED is an appropriate setting for addressing these digital equity issues. Answers to such questions will be elucidated in the currently ongoing associated study. We hope to continue to address digital health inequities by expanding our program to more sites, and by ensuring the ongoing activation of SIM cards currently in circulation.

## Appendix

Multimedia Appendix 1 PHONE-CONNECT follow-up interview guide.

## Abbreviations

ED: emergency department
PHONE-CONNECT: Phones for Healthier Ontarians iN EDs – COvid NEeds met by Cellular Telephone
UHN: University Health Network
