# Supplementary material for: Prescribing Phones to Address Health Equity Needs in the COVID-19 Era: The PHONE-CONNECT Program
Source: J Med Internet Res. 2021 Apr 6;23(4):e23914. doi: 10.2196/23914 (PMC8025912; doi:10.2196/23914)
Supplement: Multimedia Appendix 1 [file jmir_v23i4e23914_app1.docx]

**Appendix 1**

1. How has having a phone impacted your daily life?

Q1: What have you been using this phone for over the last month?

Q2: Were you tested for Coronavirus over the last month? (IF NO - Skip to Q3)

Q2b: If you were tested for Coronavirus over the last month did you use this phone to receive your test results?

Q2c: Have you been self-isolating due to COVID anytime over the last month?

Q2d: If you have been self-isolating due to COVID do you feel that having this cellphone has made it easier for you to follow self-isolation guidelines?

Q3: Did you use this phone to connect with any social services?

Q4: If you used this phone to connect with social services, which ones?

Q4b if other, specify

Q5: Have you used this phone to attend virtual follow up appointments with a health care provider?

Q6a: If yes, which ones: (free text)

Q6: How often would you say you have used this phone to speak with friends or family?

Q7: To what extent has having this cellphone impacted your wellbeing and mental health?

Q7a: Why has or hasn't this phone contributed to your well-being? (Free text)

Q8: Is there anything else you'd like to share with us regarding your experience using this phone? (Free Text)
